# Low-energy hip fracture in a socially withdrawn and physically inactive teenager highlighting the age-independent role of mechanical loading and nutrition: a case report

**DOI:** 10.1186/s13256-026-06258-7

**Published:** 2026-07-09

**Authors:** Muhammed Cetinkaya, Mathias Mejdahl, Julius Krüger, Dennis Karimi, Tobias Winkler, Tazio Maleitzke

**Affiliations:** 1 Trauma Orthopaedic Research Copenhagen Hvidovre (TORCH), Department of Orthopaedic Surgery, Copenhagen University Hospital Hvidovre, Hvidovre, Denmark; 2 Department of Orthopedic Surgery, Zealand University Hospital, Køge, Denmark; 3 Charité – Universitätsmedizin Berlin, corporate member of Freie Universität Berlin and Humboldt-Universität zu Berlin, Center for Musculoskeletal Surgery, Berlin, Germany; 4 Berlin Institute of Health at Charité – Universitätsmedizin Berlin, Julius Wolff Institute, Berlin, Germany; 5 Berlin Institute of Health Center for Regenerative Therapies,, Berlin Institute of Health at Charité – Universitätsmedizin Berlin, Berlin, Germany; 6 Department of Clinical Medicine, University of Copenhagen, Copenhagen, Denmark

**Keywords:** Adolescence, Low-energy trauma, Vitamin D deficiency, Mechanical unloading, Pediatric hip fracture, Mechanical loading, Case report

## Abstract

**Background:**

Proximal femur fractures in adolescents are rare and usually occur following high-energy trauma. When such injuries result from minimal trauma, they demand careful evaluation of skeletal integrity. This case highlights how social withdrawal, prolonged immobilization, and malnutrition can lead to reversible bone fragility and an unusual fracture pattern in a young patient.

**Case presentation:**

A 14-year-old boy of Danish ethnicity with no prior somatic disease sustained an intertrochanteric femur fracture after falling from a floor bed of approximately one meter.

For the preceding two years, he had been socially withdrawn and largely bedridden. Profound malnutrition, vitamin D deficiency, secondary hyperparathyroidism, and markedly reduced bone quality were evident. Intraoperative findings confirmed cortical thinning and brittle bone structure inconsistent with chronological age. The patient was treated with surgical fracture fixation using a pediatric dynamic hip screw in combination with vitamin D and calcium supplementation, calorie-dense nutrition, physiotherapy, and social support. The fracture healed, and over eight months he regained mobility, caught up on body weight and growth, and improved in developmental parameters.

**Conclusions:**

Low-energy hip fractures in adolescents are rare but may indicate reversible bone fragility from unloading and malnutrition; early multidisciplinary interventions are warranted.

## Background

Pediatric proximal femur fractures are very rare, representing < 1% of pediatric fractures [1, 2]. In a large binational study of 6410 surgically treated femoral fractures in children under 17 years, conducted in Finland and Sweden between 1998 and 2016, the overall annual incidence of femoral fractures was 11–13 per 100,000 children [3]. Most fractures involved the femoral shaft, while proximal and distal femur fractures were infrequent [3]. When proximal fractures occur, they are usually the result of high-energy trauma, and only about 10% are due to low-energy falls [1, 2].

Adolescence is the period of rapid skeletal growth, with 40–60% of peak bone mass (PBM) acquired during the pubertal growth spurt [4]. Achieving optimal PBM depends on a balanced diet with calcium, protein, and vitamin D, combined with weight-bearing exercise [5]. Adequate nutrition and impact activity maximize PBM and protect against fractures.

Bone adapts to habitual loading according to Wolff’s law [6]. Frost formalized this concept as the “mechanostat,” suggesting that bone mass is maintained within a physiological strain window—strains above the upper limit trigger bone modeling and net gain, while strains below the lower limit promote resorption [7]. Nutritional factors are equally important. In a pediatric cohort, vitamin D levels below 40 ng/mL were associated with higher fracture incidences, while supplementation improved callus formation in those with fractures [8].

Bones of astronauts display skeletal adaptation to unloading. Prolonged exposure to microgravity leads to significant bone loss, especially in weight-bearing bones such as the proximal femur. Despite structured in-flight exercise protocols, these changes are not fully mitigated. LeBlanc et al. conducted longitudinal measurements of bone and muscle mass using dual-energy X-ray absorptiometry before and after long-duration spaceflight and concluded that “the current in-flight exercise program is not sufficient to completely ameliorate bone and muscle loss during weightlessness” [9].

Similarly, Lang et al. used quantitative computed tomography (CT) to assess bone adaptation after long-duration spaceflight, finding that the most severe bone loss occurs in the proximal femur [10].

An intertrochanteric femur fracture following a trivial fall in a 14-year-old is highly unusual and indicates profoundly altered skeletal physiology. We present the case of a physically inactive, socially isolated, and malnourished adolescent who sustained such a fracture after falling from a floor bed, and describe his subsequent recovery of bone health, growth, and social inclusion.

## Case presentation

### Initial presentation

A 14-year-old boy of Danish ethnicity was admitted to the emergency department with right-sided hip pain after falling 1 m from his bed. There were no prior somatic diseases, medical evaluations, or interventions for nutrition, bone health, or mobility recorded before admission.

**Fig. 1:**
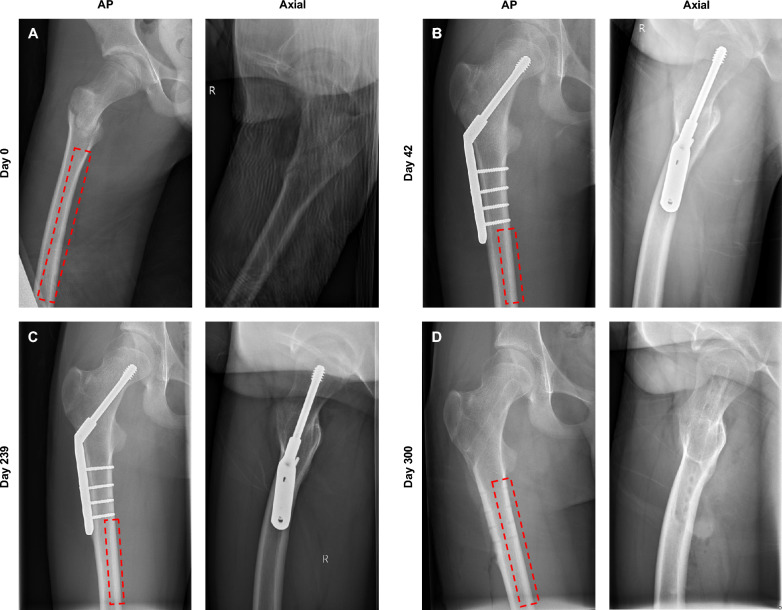
Radiographs of patient’s right hip in two planes (anteroposterior and axial) on day 0 (**A**), during the postoperative phase on day 42 (**B**) and on day 239 (**C**), and after implant removal on day 300 (**D**), with progressive increase in cortical thickness (red boxes)

Examination revealed bilateral equinus contractures and radiographs showed a displaced intertrochanteric femur fracture (Fig. 1A). The patient weighed 36 kg, was 160 cm tall (body mass index 14.1), and appeared clinically malnourished.

### Social context

The patient, who had previously been diagnosed with attention deficit hyperactivity disorder and described as taciturn, had missed 2½ years of formal school and instead received homeschooling with outreach teaching 3–4 times weekly due to severe school refusal following bullying episodes.

This prolonged absence from formal schooling and associated social withdrawal were attributable to multiple interacting factors including underlying attention deficit hyperactivity disorder, repeated bullying at school which was the primary driver, insufficient continuity in social services follow-up, and progressive excessive computer game use leading to a predominantly sedentary lifestyle.

He spent most of his time in bed or sitting at his computer, playing video games for most of his waking hours. Meals were often served by his mother at the desk. The patient’s parents were divorced, and he mainly lived with his mother in a fifth-floor apartment with an elevator.

### Surgery

On day 2, definitive fracture fixation was achieved with a pediatric dynamic hip screw (Smith & Nephew). Intraoperative fluoroscopy revealed severe osteopenia with cortical thinning and brittle bone inconsistent with the patient’s chronological age (Fig. 1B).

Adolescent intertrochanteric fractures can in principle be treated with intramedullary nailing, different (pediatric) plating systems, or the dynamic hip screw. As this is a rare injury, comparative studies do not exist and available datasets are underpowered for comparison and often pool various proximal femur fracture types [20, 21]. As the fracture was relatively stable in our case, the dynamic hip screw system was chosen.

### Admission (day 0–56)

#### Nutrition, vitamins, biochemistry, and body weight

Baseline tests indicated nutritional rickets: 25-hydroxy-vitamin D < 12 nmol/L, secondary hyperparathyroidism (parathyroid hormone 28.6 pmol/L), ionized calcium 1.19 mmol/L, alkaline phosphatase 370 U/L, and zinc 8 µmol/L. A baseline DXA scan was not performed as the acute surgical treatment and nutritional support were prioritized following admission.

**Fig. 2:**
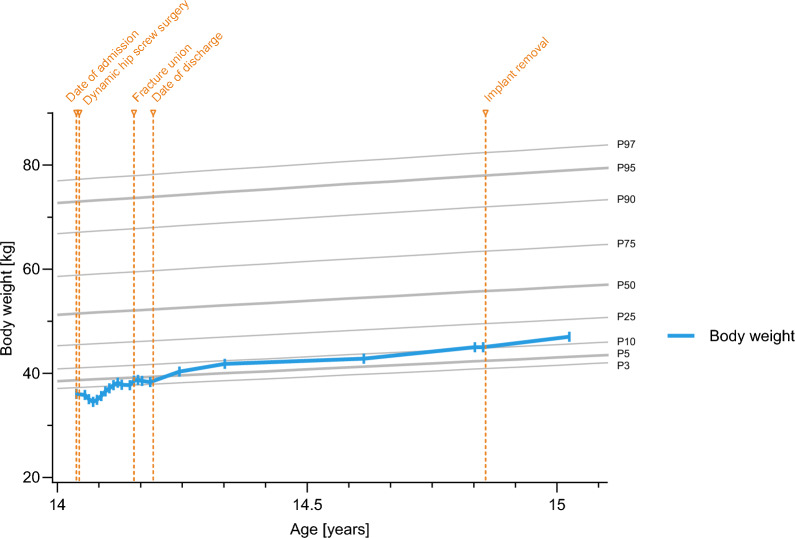
Patient’s body weight over time, compared with United States Center for Disease Control and Prevention body weight-for-age percentile curves [19]. Sequential body weight measurements increased from 34.9 kg (day 15) to 35.6 kg (day 18), 36.5 kg (day 22), 37.1 kg (day 25), 37.8 kg (day 35), 38.6 kg (day 43), and 38.7 kg (day 46). P = percentile

Phytomenadione was given intramuscularly as a single 10 mg dose for elevated INR consistent with vitamin K deficiency secondary to malnutrition, followed by 12 days of oral colecalciferol loading for severe vitamin D deficiency and nutritional rickets (200,000 IU once, followed by 20 µg daily) and daily UniKalk Forte^®^ as calcium supplementation also for nutritional rickets (400 mg four times daily for 3 days, then 400 mg twice daily) combined with multivitamins. A dietitian initiated a calorie-dense diet with fluid balance monitoring. Body weight rose gradually from 34.9 to 38.7 kg during admission (Fig. 2).

#### Physiotherapy and ergotherapy

Mobilization began on day 3; by day 5 the patient used a wheelchair, walked 24 m with a frame on day 8, and used a forearm-support walker on day 11. Hydrotherapy was introduced on day 19. By day 26 the patient could walk short distances with elbow crutches, progressing to full weight-bearing with a walking aid on day 43. On day 54 he walked ~ 500 m with forearm crutches. Occupational therapy focused on daily activities such as dressing, showering, and tooth-brushing, which was previously done by his mother.

#### Social and school developments

Three multidisciplinary meetings (parents, case manager, nursing staff, physicians, social worker) were held on days 7, 40, and 55. No formal psychiatric assessment was conducted during hospitalization. The parents and social services were advised about the option of further ambulatory psychiatric support following admission. The patient exhibited severe school refusal and social withdrawal, spending most time playing computer games, with limited food intake, physical inactivity, and high dependence on his mother. Initially he resisted physiotherapy, schooling, and structured routines, and reacted harshly when screen time was restricted. With close support and consistent routines, he gradually engaged in physiotherapy, improved nutrition, and accepted short periods of hospital-based schooling. Both parents received guidance on routines, nutrition, pain management, and screen time limits. A structured daily schedule with hospital schooling, physiotherapy, and restricted screen time was established. He was discharged on day 56.

### Pediatric follow-ups and rehabilitation (day 56–299)

No adverse or unanticipated events occurred.

#### Nutrition, vitamins, biochemistry, and body weight

By day 116, blood tests were normal, and only multivitamins were continued.

Low vitamin B₁₂ (164 pmol/L) was treated with Betolvex^®^ (hydroxocobalamin 1 mg intramuscularly). Body weight and height increased from 40.3 kg/170 cm on day 77 to 45 kg/178 cm on day 291 (Fig. 2).

#### Physiotherapy and ergotherapy

On day 77, the patient used a single elbow crutch and lacked 3 cm dorsiflexion in both ankles. Daily physiotherapy targeted strength, endurance, balance, and core stability. The patient’s gait was still affected on day 89, but by day 239 the equinus deformity had markedly improved following Achilles stretching. Occupational therapy twice weekly addressed self-care and meal preparation, and by day 225 he attended a public gym three times weekly.

#### Social and school developments

Home schooling continued for 2 h daily after discharge and until day 238, when he transitioned to a special education institution. A youth mentor organized outdoor activities five afternoons weekly, and computer games were limited to 4–4.5 h daily.

### Orthopedic follow-up and implant removal

Although rickets was confirmed by laboratory tests, hand and knee radiographs were normal on day 5. Fracture union was confirmed on day 42 (Fig. 1B). The implant was removed on day 239 (Fig. 1C), and postoperative radiographs showed restored femoral anatomy (Fig. 1D). Full weight-bearing was allowed immediately after implant removal.

## Discussion

Low-energy hip fractures in adolescence are rare, and an intertrochanteric fracture in a 14-year-old presumably healthy patient after a 1 m fall indicates markedly altered bone metabolism. Clinical, radiological, and biochemical examinations revealed bilateral equinus contractures, cortical thinning, and malnutrition following years of social isolation.

At admission, the patient was underweight, vitamin D-deficient, and had hyperparathyroidism, largely due to prolonged inactivity and limited sunlight exposure.

Comparable cases are rarely reported. Rajendran et al. described a 14-year-old boy with a low-energy femoral neck fracture during COVID-19 lockdown, where confinement, poor diet, and low sunlight exposure caused severe vitamin D deficiency. After fracture fixation, nutrient supplementation, and structured rehabilitation, the patient reportedly recovered fully [11].

Similarly, vitamin D insufficiency (20–30 ng/mL) is associated with an approximately threefold higher fracture risk, as shown in a prospective case–control study of 120 children, after adjusting for age and daily sun exposure [12]. A Romanian cohort of 1577 children showed a 2.1-fold increased fracture risk when levels were below 30 ng/mL, independent of age and sex [8].

In our patient, inactivity eliminated bone-loading stimuli and undernutrition deprived the skeleton of essential substrates, resulting in cortical thinning consistent with Wolff’s law [6] and Frost’s “mechanostat” [7].

Evidence supports this mechanism: a 2024 meta-analysis of 15 randomized controlled trials (RCTs) (*n* = 723, ages 10–19) found that weight-bearing exercise increased bone mineral density at the spine (standardized mean difference [SMD] = 0.34) and femoral neck (SMD = 0.31) [13]. A 10% increase in peak PBM may delay osteoporosis onset by 13 years in women [14].

Once the femur was surgically stabilized in our patient and nutrition was restored, the “mechanostat” shifted toward modeling. Body weight rose from 34.9 to 45 kg, vitamin D normalized to 58 nmol/L, and height increased by 18 cm. This rapid recovery highlights the plasticity of the adolescent skeleton when both substrates and loading are restored.

Nutrient repletion alone, however, is insufficient. In an RCT of 8851 Mongolian schoolchildren, high-dose vitamin D for three years did not reduce fracture incidence [15].

The authors did not report data on physical activity; thus, it can be inferred that only biochemical sufficiency, combined with adequate mechanical loading, leads to structural resilience. Alternatively, these children might have had normal bone health at baseline, meaning vitamin D supplementation alone could not further improve bone quality.

This case illustrates a broader principle: skeletal robustness depends on the continuous interplay between biological factors and mechanical loading, both of which are influenced by the social environment. Low-energy fractures in adolescents may therefore result not from chronological age alone, but from a combination of physical inactivity, inadequate nutrition, and limited sunlight exposure. When this interaction is disturbed—whether by microgravity in space [9, 10], prolonged immobility in older adults, or sedentary lifestyles in adolescents—the skeleton adapts, increasing fracture risk.

Beyond surgical fixation and medical management, our case highlights the importance of structured social reintegration. During hospitalization, a multidisciplinary team introduced daily routines, hospital schooling, and limited screen time, while occupational therapy promoted independence in daily activities. After discharge, home schooling was gradually transitioned to a special education setting, and a youth mentor facilitated outdoor activities several times per week, counteracting prolonged social isolation. Combined with physiotherapy, nutritional support, and medical care, these measures not only restored physical health but also reversed extended social withdrawal.

Research supports the influence of psychosocial factors on bone health. Chan et al. reviewed 34 studies showing that family and peer support, self-efficacy, and team sports participation were associated with bone-protective behaviors and consequently with higher bone mineral density and bone mineral content [16]. Heath et al. demonstrated in 395 children with long-bone fractures that lower socioeconomic status delayed union by ~ 10 days per $10,000 median household income decrease [17]. Furthermore, in an RCT of 40 elderly hip-fracture patients, structured psychosocial counseling improved health-related quality of life and reduced anxiety, depression, and postoperative pain compared to standard care [18].

Taken together, these findings emphasize that effective management of bone fragility requires not only medical and surgical strategies but also structured psychosocial and educational interventions tailored to the individual patient.

This case provides detailed longitudinal clinical, biochemical, and functional data on a rare low-energy intertrochanteric femur fracture in an adolescent managed with a multidisciplinary treatment approach. Limitations include the single case design, absence of baseline bone mineral density assessment, lack of objective pre injury physical activity data, absence of standardized functional outcome measures, and no long-term post-healing assessment of bone density, which limits generalizability.

## Conclusion

Skeletal fragility is not inherently age related but arises when mechanical loading and nutrition are insufficient. This case illustrates that principles of bone adaptation and deconditioning apply across the lifespan. Addressing social withdrawal in teenagers with low-energy fractures may help guide multimodal treatment in such rare cases.

## Data Availability

All data generated and analyzed during this study are included in this published article. No additional datasets were generated. Further anonymized details are available from the corresponding author on reasonable request, in line with institutional and national regulations.

## Abbreviations

m: Meter
PBM: Peak bone mass
RCT: Randomized controlled trial
SMD: Standardized mean difference
